# Supplementary material for: Agent-Based and Continuum Models for Spatial Dynamics of Infection by Oncolytic Viruses
Source: Bull Math Biol. 2023 Aug 31;85(10):92. doi: 10.1007/s11538-023-01192-x (PMC10471645; doi:10.1007/s11538-023-01192-x)
Supplement: Supplementary file 1 — (pdf 122 KB) [file 11538_2023_1192_MOESM1_ESM.pdf]

Description of Electronic Supplementary Material for 'Agent-Based and Continuum Models for Spatial Dynamics of Infection by Oncolytic Viruses'

David Morselli, Marcello Edoardo Delitala, Federico Frascoli

Online Resource S1 (this file)

Description of the videos in the rest of the Electronic Supplementary Material.

Online Resource S2

Video to support Fig. 3 in the paper, showing the comparison in one spatial dimension between numerical simulations of the discrete model with undirected movement (solid lines) and the numerical solution of Eq. (2.4) (dotted black lines) with the parameters given in Table 1. For the agent-based model, the density of the uninfected cells is represented in blue and the density of infected cells in red. The vertical dashed lines represent the expected positions of the uninfected and infected invasion fronts, traveling respectively at speed $2\sqrt{D_u p}$ (blue lines) and $2\sqrt{D_i(\beta - q)}$ (red lines); the latter has no biological meaning at late times, as the infection cannot go beyond the uninfected front. The horizontal solid black lines show the equilibrium of the ODE given by Eq. (3.4) and the horizontal dashed yellow line represents the expected uninfected density at the front given by Eq. (3.5) (only relevant at late times). The results of the agent based model are averaged over five simulations and the maximum of the cell density axis corresponds to the maximum over time of this average (which is lager than the carrying capacity).

Online Resource S3

Video to support Fig. 4 in the paper, showing the comparison in two spatial dimensions between numerical simulations of the discrete model with undirected movement and the numerical solution of Eq. (2.4) with the parameters given in Table 1. The dotted green circles represent the internal minimum of the numerical solution of Eq. (2.4) (not shown when this minimum is in 0). The dashed cyan circles represent the expected positions of the uninfected and infected invasion fronts, traveling respectively at speed $2\sqrt{D_u p}$ and $2\sqrt{D_i(\beta - q)}$; the latter has no biological meaning at late times, as the infection cannot go beyond the uninfected front. The dashed red circles represent the front of the infected cells given by the numerical solution of Eq. (2.4). The results of the agent based model are averaged over five simulations and the maximum of the colorbars for uninfected and infected cells correspond to the maximum over time of the averages (which for the uninfected cells is lager than the carrying capacity).

Online Resource S4

Video to support Fig. 6a in the paper, showing the comparison in two spatial dimensions between numerical simulations of the discrete model with undirected movement and exponential growth and the numerical solution of the equation

$$\begin{cases} \partial_t u(t,x) = D_u \partial_{xx}^2 u(t,x) + pu(t,x) - \dfrac{\beta}{K} u(t,x) i(t,x) \\ \partial_t i(t,x) = D_i \partial_{xx}^2 i(t,x) + \dfrac{\beta}{K} u(t,x) i(t,x) - qi(t,x) \end{cases}$$

with the parameters given in Table 1. The dashed cyan circles represent the expected positions of the uninfected invasion fronts, traveling at speed $2\sqrt{D_u p}$. The dashed red circles represent the front of the infected cells given by the numerical solution of the PDE. The results of the agent based model are averaged over five simulations. The maximum of the colorbars changes at time $t = 180$ h to better adapt to the different growth phases.

Online Resource S5

Comparison in one spatial dimension between numerical simulation of the discrete model with pressure-driven movement (solid lines) and the numerical solution of Eq. (2.5) (dotted black lines). For the agent-based model, the density of the uninfected cells is represented in blue and the density of infected cells in red. The vertical dashed blue lines represent the position of the uninfected invasion fronts if it were traveling at speed $\sqrt{D_u p/2}$; observe that the actual speed is smaller. The horizontal solid black lines show the equilibrium of the ODE given by Eq. (3.4). The parameters employed are the ones given in Table 1, with the exception of the diffusion coefficients D_u, D_i (which are set to 3.00×10^{-1} mm^2/h, i.e. twenty times the reference value), the carrying capacity K (which is set to 10^5 cells/mm, i.e. one hundred times the reference value) and the death rate of infected cells q (which is set to 4.17×10^{-3} h^{-1}, i.e. one tenth of the reference values). This parameter choices allow a perfect agreement between the discrete and the continuous model, but appears biologically unrealistic. The results of the agent based model are averaged over five simulations.

Online Resource S6

Video to support Fig. 9 in the paper, showing the comparison in two spatial dimensions between numerical simulations of the discrete model with pressure-driven movement and the numerical solution of Eq. (2.5). The dotted green circles represent the internal minimum of the numerical solution of Eq. (2.5) (not shown when this minimum is in 0). The dashed cyan circles represent the expected positions of the uninfected invasion fronts in absence of treatment, traveling at speed $\sqrt{D_u p/2}$. The dashed red circles represent the front of the infected cells given by the numerical solution of Eq. (2.5). The parameters employed are the ones given in Table 1, with the exception of the carrying capacity K (which is set to 10^5 cells/mm^2, i.e. ten times the reference value) and the death rate of infected cells q (which is set to 8.33×10^{-3} h^{-1}, i.e. one fifth of the reference values). This parameter choices allow a perfect agreement between the discrete and the continuous model. The results of the agent based model are averaged over five simulations and the maximum of the colorbars for uninfected and infected cells correspond to the maximum over time of the averages.
